# Non-Official Hospitals and the British Red Cross Society

**Published:** 1914-12-05

**Authors:** 


### The Hospital

The Workers' Newspaper of Administrative Medicine and Institutional Life, Administration, National Insurance and Health.

No. 1485, Vol. LVII. SATURDAY, DECEMBER 5, 1914. PRICE ONE PENNY.

### NON-OFFICIAL HOSPITALS AND THE BRITISH RED CROSS SOCIETY.

In a letter published in the *Morning Post* of the 25th ult., Sir George Beatson, writing as a member of the British Red Cross Council, utters some grave warnings of the disadvantages and risks which are attached to non-official attempts to provide hospital treatment for our sick and wounded soldiers. Starting with the proposition that the provision of such treatment is primarily the duty of the naval and military authorities, he claims that any assistance these may need should be furnished exclusively through the British Red Cross organisation; and he urges the public to withhold support from all private schemes which do not enjoy official countenance and sanction.

For the position thus presented Sir George gives reasons which deserve attention, both on their own merits and in virtue of the authority on which they rest. In the first place, Sir George offers the assurance, speaking as a surgeon who has recently visited the war area in France, that the British sick and wounded in the military hospitals are receiving "the very best of treatment and care"; and he speaks in high praise of the surgical efficiency of the Royal Army Medical Corps. Next he contends that private enterprise in the matter now in question means a waste of money and the multiplication of unnecessary appeals to the public, with inevitable confusion and loss of force. And lastly he warns us that these unauthorised schemes, lacking, as they do, official supervision and control, afford opportunities for the work and activities of spies. On this last-mentioned point Sir George suggests that the large and varied *personnel* sent out to the seat of war in connection with hospital or ambulance organisations may readily include undesirable and mischievous individuals, who may easily gain information of the dispositions of the fighting forces and may utilise such information for unpatriotic ends. To prevent such disasters authoritative investigation is essential, and in this respect, we are told, "regrettable laxity" has already marked some of the unofficial efforts. In short, the purpose of the letter now before us is to announce that private hospital and other unsanctioned proposals are unnecessary and uneconomical, and that they offer ready and frequent opportunities for the practice of espionage. The obvious remedy for these ills and dangers is that public support shall be given only to the British Red Cross Society as the authorised agency through which the War Office makes known its wants, and as the sole organisation by which economical management and efficient inspection can be guaranteed. Until the Red Cross Society has proclaimed what effort is desired it will be well for the public to do nothing, or at least to abstain from lending any assistance and support to the proposals of private individuals and self-constituted committees.

We recognise, and our readers will recognise, that any opinion expressed by Sir George Beatson, and dealing with the surgical care of wounded soldiers, ought to command the respect and attention of all reasonable persons. Sir George is not only an experienced surgeon; he has also for many years devoted both time and ability to the advance of military medical organisation, and on the subject of his letter he speaks as one having first-hand knowledge. Further, we will venture to say that with much of what he writes most people will find themselves in hearty agreement. Obviously it is not wise that without effective control any organisation calling itself a hospital or other philanthropic name should be allowed to proceed to the seat of war and to take charge of sick and wounded soldiers. These men are the care of the nation, and it is due to them that they shall be placed in the hands of those who are known to be competent by the nation's representatives. Equally it is not wise that some few individuals, with the aid perhaps of certain friendly medical practitioners, shall be allowed to appeal for a favoured hospital scheme at the risk of diverting support from enterprises which are both essential and efficient. And while there is, perhaps, just now a danger that the word "spy" may be overdone, it is impossible to doubt that Sir George Beatson would never have written what he has written without substantial and abundant warrant. This consideration alone, and apart from all others, is more than sufficient to justify the refusal of the War Office to recognise any hospital which does not submit itself to the conditions and control of the British Red Cross Society.

There is one other point on which Sir George Beatson will command all our suffrages. It is the recognition he gives to the earnest and benevolent intentions both of the promoters and of the supporters of the schemes which he condemns. He wishes to sustain these motives, but to direct them into wise channels. The end which all desire can be obtained with the maximum of efficiency and economy only by affording adequate and united support to the official apparatus of the British Red Cross Society. This is the sum and substance of Sir George Beatson's argument, and, at least in the main, we are decidedly of his opinion, and on the practical plane we heartily endorse his advice. It seems reasonable to ask why advice, which can defend its value by such common-sense reasons, should be necessary; and why, openly and conspicuously, it has failed to secure general assent. The answer we believe to be that, with all its virtues, the British Red Cross Society has not succeeded in securing for itself on a widespread and extensive scale the confidence and sympathy of the nation. If a central organisation wishes both to keep authority and at the same time to command widespread support it must learn to take note of local feeling and popular wishes, and must not remain isolated and detached on some pedestal of superiority.

